# Family Strength, Disability Acceptance, and Life Satisfaction in Older Adults With Hearing Loss

**DOI:** 10.1093/geroni/igaf122.4118

**Published:** 2025-12-31

**Authors:** Seohyeon Kim, Sunghee Tak

**Affiliations:** Seoul National University, Seoul, Republic of Korea; Seoul National University, Seoul, Republic of Korea

## Abstract

Family strength is considered a key contributor to life satisfaction among individuals with disabilities. However, little is known about its longitudinal influence on the older adults with hearing loss. This study aimed to examine the long-term effects of family strength on life satisfaction and to investigate the moderating role of disability acceptance. Data were drawn from the Disability and Life Dynamics Panel in South Korea, spanning six waves from 2018 to 2023. A total of 437 participants aged 50 years and above with registered hearing disabilities were included in the analysis. Generalized Estimating Equations (GEE) were employed to analyze the overall associations between main variables across repeated measures, and Group-Based Trajectory Modeling (GBTM) was applied to capture distinct subgroups with different trajectories of disability acceptance over time. At the result, three distinct trajectories emerged: “delayed improvement” (23.3%), “stable moderate” (61.7%), and “initial high then declining” (15.0%). Significant differences across groups were observed in levels of disability acceptance, family strength, and life satisfaction. GEE analysis revealed that higher family strength, educational attainment, adequate sleep duration, and employment were positively associated with life satisfaction. However, the moderating effect of disability was not statistically significant. These findings highlight the importance of the family-based determinants in life satisfaction in individuals with sensory impairments. Therefore, a tailored approach that fosters family support and acknowledges varying trajectories of disability acceptance may be beneficial in promoting well-being among the older adults with hearing impairments.

